# MicroRNA-33a-5p sponges to inhibit pancreatic β-cell function in gestational diabetes mellitus LncRNA DANCR

**DOI:** 10.1186/s12958-020-00618-8

**Published:** 2020-06-06

**Authors:** Yan Feng, Xin Qu, Yu Chen, Qi Feng, Yinghong Zhang, Jianwei Hu, Xiaoyan Li

**Affiliations:** 1 Department of Clinical Nutrition, Yuhuangding Hospital Affiliated to Qingdao University, No. 20 East Yuhuangding Road, Yantai, 264000 Shandong China; 2 Department of Obstetrics and Gynecology, Yuhuangding Hospital Affiliated to Qingdao University, No. 20 East Yuhuangding Road, Yantai, 264000 Shandong China; 3 Department of Gynecology, Penglai People’s Hospital, No. 89, Xianhou Road, Penglai, 265600 Shandong China; 4 Department of General Surgery, CPLA No. 71897, No. 1 Bayi Road, Xi’an, 710000 Shaanxi China; 5 Department of Group Health, Maternal and Child Health Institution, Kunshan, 215301 Jiangsu China

**Keywords:** GDM, DANCR, miR-33a-5p, ABCA1, β-Cell

## Abstract

**Background:**

Gestational diabetes mellitus (GDM) is the most common medical complication associated with pregnancy, which may impose risks on both mother and fetus. Micro RNAs (miRNAs) and long noncoding RNAs (lncRNAs) are implied as vital regulators in GDM. A recent paper revealed dysregulation of miR-33a-5p in placental tissues of GDM patients. However, the biological function of miR-33a-5p in GDM remains elusive. This study focused on exploring the function and underlying mechanisms of miR-33a-5p in GDM.

**Methods:**

12 GDM pregnancies and 12 healthy pregnancies were enrolled in the study. INS-1 cell line was applied in in vitro experiments. The expression levels of miR-33a-5p, lnc-DANCR (Differentiation Antagonizing Non-Protein Coding RNA), and ABCA1 (ATP-binding cassette transporter 1) mRNA were determined by RT-qPCR assay. Glucose and insulin levels were measured by ELISA assay. Luciferase reporter assay and western blot assay were applied to validate the target of miR-33a-5p.

**Results:**

miR-33a-5p was upregulated in the blood samples from GDM, and was positively correlated with blood glucose (*p* < 0.0001). Overexpression or inhibition of miR-33a-5p significantly inhibited or promoted cell growth and insulin production of INS-1 cells (*p* < 0.01). Furthermore, ABCA1 is a direct target of miR-33a-5p, and lnc-DANCR functions as a sponge for miR-33a-5p to antagonize the function of miR-33a-5p in INS-1 cells.

**Conclusion:**

Our study demonstrated that lnc-DANCR-miR-33a-5p-ABCA1 signaling cascade plays a crucial role in the regulation of the cellular function of INS-1 cells.

## Background

Gestational diabetes mellitus (GDM) is a disease that women without diabetes have a high level of blood glucose during pregnancy [1]. GDM usually occurs during the last 3 months of pregnancy and can affect pregnant women worldwide [2]. Women with GDM are at the increased risk of development of type 2 diabetes, cardiovascular disease, and obesity [3, 4]. GDM is associated with an increased frequency of fetal macrosomia, fetal morbidity, and mortality [5, 6]. Thus, a better understanding of GDM pathogenesis is critical for the development of therapeutic strategies for GDM management.

MicroRNAs (miRNAs) are small non-coding RNAs composed of 20–24 nucleotides, which can bind to the sequence site of target mRNA at 3′-untranslational (UTR) region, causing degradation of mRNA or inhibition of protein translation [7]. MiRNAs are recognized as vital regulators in various human diseases pathogenesis and progression [8], including GDM [9, 10]. MiRNAs participate in the regulation of GDM by affecting insulin production or endocrine pancreas development. For example, miR-9, an abundantly expressed miRNA in pancreatic islets, decreased the beta-cell secreted insulin upon glucose stimulation. MiR-9 regulates insulin production through down-regulation of Onecut2 (one cut domain, family member) and/or up-regulation of Rab (ras-related GTP-binding protein) [11]. MiR-7, a highly expressed miRNA in islets and pancreas, targets paired box 6 (Pax 6) to negatively regulate beta cells differentiation [12, 13].

Accumulating evidence links miR-33a-5p to cholesterol homeostasis and fatty acid/glucose metabolism. For example, several groups reported that miR-33a-5p regulates the efflux of cholesterol to lipid-poor apolipoprotein A-I (apo A-I) and forms nascent high-density lipoprotein (HDL) by targeting ATP-binding cassette transporter 1 (ABCA1), and the latter is involved in HDL biogenesis and reverse cholesterol transport [14–16]. Furthermore, miR-33a-5p was shown to target ABCA1 to modulate cholesterol accumulation and insulin secretion [17]. Similarly, multiple papers reported that miR-33a-5p targets ABCA1 in atherogenesis [18], morbidly obese [19], and brain [20]. Furthermore, miR-33a-5p was found to mediate fatty acid metabolism and insulin signaling by targeting the insulin receptor substrate 2 (IRS2) [21]. Furthermore, miR-33a-5p was implied to play pivotal roles in the regulation of glucose metabolism to affect aging-related insulin resistance and adiposity as well as radio-sensitivity of melanoma [22, 23]. Despite various metabolic-regulation effects of miR-33a-5p have been demonstrated in multiple human diseases, the functional role of miR-33a-5p in GDM is still lacking. In 2015, Li et al. performed a miRNA microarray analysis on 30 placental samples from 15 GDM patients and 15 healthy donors, and they revealed miR-33a-5p to be potentially involved in the induction of fetal macrosomia, a GDM-related complication [24]. However, whether miR-33a-5p is indeed involved in the regulation of GDM needs further confirmation. The underlying molecular mechanism of the miR-33a-5p-mediated biological function of beta cells remains unknown.

In the current study, we focused on investigating the biological relevance of miR-33a-5p in the regulation of GDM and its potential regulation mechanism using a rat insulinoma cell line, INS-1 cell, which is a well-established cellular model for islet beta-cell function study. We aimed to address the following questions: 1. whether miR-33a-5p is dysregulated in GDM; 2. What is the potential target of miR-33a-5p that may be associated with its function; 3. Which lnc RNA is responsible for the regulation of miR-33a-5p; 4. What is the effect of miR-33a-5p on cell proliferation and insulin production of INS-1 cells?

## Materials and methods

### Patients’ specimens

In this study, 12 pregnancies with GDM and 12 healthy pregnancies (control) were chosen to participate. All participants underwent a 75-g oral glucose tolerance test (OGTT) between weeks 24 and 28 of their pregnancy. The criteria of the Chinese society for Diabetes Mellitus (capillary whole blood; fasting glucose > 90 mg/dL, 1 h > 180 mg/dL, and 2 h > 155 mg/dL) was used, and the diagnosis GDM was given when two measurements were above limits. And the mean birth weight in the GDM was 3787.4 g, and in the control group was 3320.8 g. In each group, fetal gender was balanced. Fetal gender was balanced between two groups. The samples were collected from Yuhuangding Hospital Affiliated to Qingdao University and snapped frozen in − 80 °C for further analysis. Written informed consent was obtained from each participant prior to sample collection. The study protocol was approved by the Ethics Committee of Yuhuangding Hospital Affiliated to Qingdao University.

### Cell culture

HEK293 cell line, a permanent cell line established from primary embryonic human kidney, was obtained from American Type Culture Collection (ATCC, Manassas, USA) and INS-1 cell line was purchased from ThermoFisher scientific (MD, USA). The cultures were maintained in Dulbecco’s modified Eagle’s medium (Thermo Fisher Scientific, Waltham, MA, USA) supplemented with 10% fetal bovine serum (Thermo Fisher Scientific), penicillin (100 U/ml) and streptomycin (100 U/ml) (Sigma-Aldrich, St. Louis, MO, USA) at 37 °C in a humidified incubator with 5% CO_2_.

### Quantitative reverse transcription-PCR (RT-qPCR)

Total RNA was extracted by using TRIzol reagent (Invitrogen, Carlsbad, CA, USA), and the RNA concentration and purity were determined by NanoDro2000c spectrophotometer (Thermo Scientific, Waltham, USA). Reverse transcription was performed using TaqMan MicroRNA Reverse Transcription Kit (Applied Biosystems, Carlsbad, CA). The RT-qPCR process was performed on an ABI7500 Real-time PCR system.

### Western blot

Cells were lysed in a RIPA buffer (PI89901, Thermo Scientific Pierce, MD, USA) supplied with a protease inhibitor cocktail (A32963, Thermo Scientific Pierce, MD, USA). A total of 20 μg of proteins were loaded to 10% SDS-polyacrylamide gel and separated by electrophoresis. The proteins were transferred onto polyvinylidene difluoride (PVDF, Bio-Rad, Hercules, CA, USA) membranes followed by blocking with 5% non-fat milk and incubating with primary antibodies against ABCA1 or GAPDH (Cell Signaling Technology, Danvers, MA, USA). Then, the membranes were incubated with HRP-conjugated secondary antibodies. Signals were visualized by using ECL (Millipore, Burlington, MA, USA).

### Enzyme-linked immunosorbent assay (ELISA)

The blood glucose levels were measured using a glucose assay kit (ab65333) from Abcam (Cambridge, MA, USA), and insulin secretion levels were determined using an insulin ELISA kit from RayBiotech (Norcross, GA, USA) according to manufactory’s instruction.

### Luciferase reporter assay

The 3′-UTR DNA fragment of wild-type or mutant ABCA1 was cloned into the psiCHECK2 reporter (Promega, WI, USA) as psiCHECK2-ABCA1-WT or psiCHECK2-ABCA1-Mut. The lnc-DANCR wild-type or mutant DNA fragment was also cloned into the psiCHECK2 reporter as psiCHECK2-DANCR-WT or psiCHECK2-DANCR-Mut.

HEK293 cells were co-transfected with miR-33a-5p mimic and psiCHECK2-ABCA1-WT, psiCHECK2-ABCA1-Mut, psiCHECK2-DANCR-WT or psiCHECK2-DANCR-Mut as well as pRT-TK Renilla plasmid. Luciferase activity was measured with the Dual-Luciferase Reporter Assay System (Promega, WI, USA) after 48 h of co-transfection.

### Cell counting Kit-8 (CCK-8) assay

Cell viability was determined by CCK8 assay according to the manufactory’s instruction (Abcam, Cambridge, MA, USA). INS-1 cells were seeded in 96-well plates and incubated for desired time points at 37 °C after transfection. The CCK-8 solution was added to each well of the plate followed by incubation at 37 °C. Absorbance was measured at 450 nm by using a microplate reader.

### Statistical analysis

Statistical analysis was performed with SPSS 13.0 software (IBM, Armonk, New York, USA). The results were evaluated by χ^2^ test, Student’s t test, or one-way ANOVA with a Tukey’s post hoc test and expressed as mean ± SD. Independent experiments were repeated at least in triplicate. The correlations were analyzed using Spearman’s correlation coefficient test. The differences were considered significant at *p* < 0.05.

## Results

### miR-33a-5p and blood glucose levels were positively correlated in GDM

**Fig. 1:**
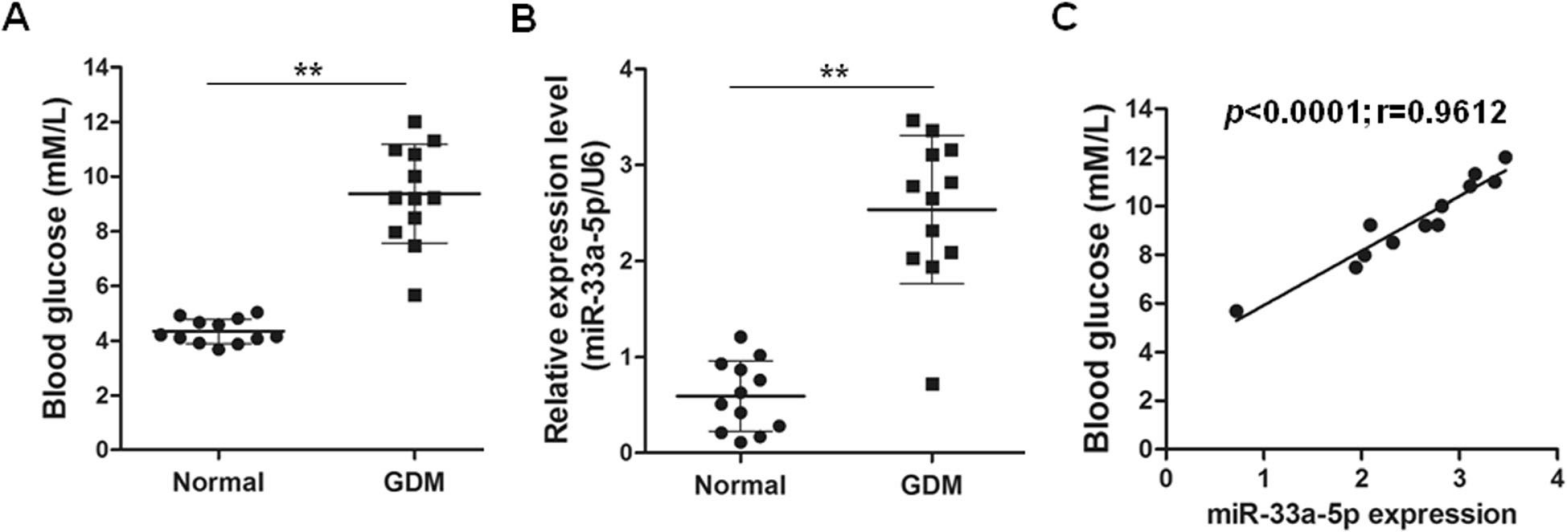
miR-33a-5p is elevated in GDM and its expression is positively correlated with blood glucose concentration. **a** The blood glucose concentration of GDM pregnancies (*n* = 12) and healthy pregnancies (*n* = 12) were analyzed. **b** The expression levels of miR-33a-5p in peripheral blood samples from GDM pregnancies (*n* = 12) and healthy pregnancies (*n* = 12) were validated by qRT-PCR. **c** Correlation between miR-33a-5p level and blood glucose was determined by Spearman correlation analysis (*n* = 12). GDM, gestational diabetes mellitus. ^**^p < 0.01

Twelve blood samples from normal healthy pregnancies and 12 blood samples from GDM pregnancies were applied in the current study. We first measured the blood glucose levels in these samples and confirmed that the blood glucose levels in GDM pregnancies were significantly higher than those in healthy donors (*p* < 0.01) (Fig. 1a). To address the question of whether miR-33a-5p is involved in GDM, we compared the expression levels of miR-33a-5p between the two groups, and we found that the serum levels of miR-33a-5p were markedly upregulated in GDM compared with those in healthy donors (*p* < 0.01) (Fig. 1b). Furthermore, Spearman correlation analysis results showed that miR-33a-5p and blood glucose levels were positively correlated in the blood samples from GDM (*p* < 0.0001; *r* = 0.9612) (Fig. 1c).

### miR-33a-5p overexpression inhibited cell proliferation and insulin production of INS-1

**Fig. 2:**
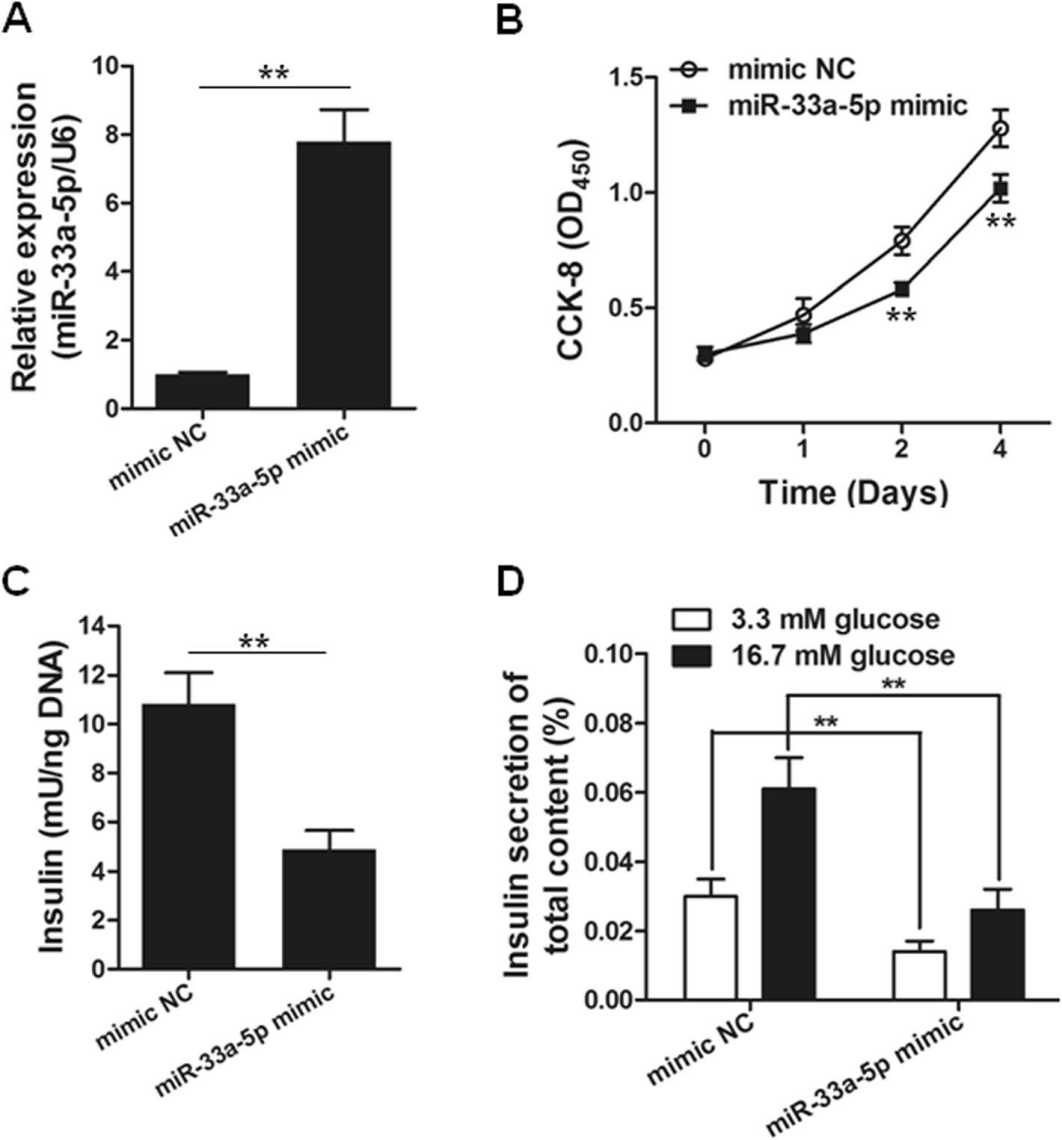
Overexpression of miR-33a-5p inhibits the functions of pancreatic β cells. **a** MiR-33a-5p mimic and mimic NC were transfected into INS-1 cells for 48 h, followed by qRT-PCR. *n* = 3. **b** INS-1 cells were transfected with miR-33a-5p mimic or mimic NC for indicated time, followed by CCK-8 assay. *n* = 6 for each time point. MiR-33a-5p mimic and mimic NC were transfected into INS-1 cells for 48 h. Insulin content **(c)** and insulin secretion **(d)** were determined by ELISA assay. *n* = 6. ^**^p < 0.01

To investigate the functional relevance of miR-33a-5p in GDM, we aimed to study the effect of miR-33a-5p overexpression on the cell proliferation and insulin production of INS-1 cells. INS-1 cells were transfected with miR-33a-5p-mimic and miR-negative control (NC) mimic. Enhanced miR-33a-5p levels in INS-1 cells after miR-33a-5p-mimic transfection were confirmed by RT-qPCR assay (Fig. 2a). The cell proliferation results showed that miR-33a-5p upregulation decreased the cell growth rate of INS-1 cells (Fig. 2b). We also observed that miR-33a-5p overexpression resulted in reduced insulin concentration as well as lowered insulin secretion in both low (3.3 mM) and high (16.7 mM) glucose conditions (Fig. 2c and d).

### miR-33a-5p suppression promoted cell proliferation and insulin production of INS-1

**Fig. 3:**
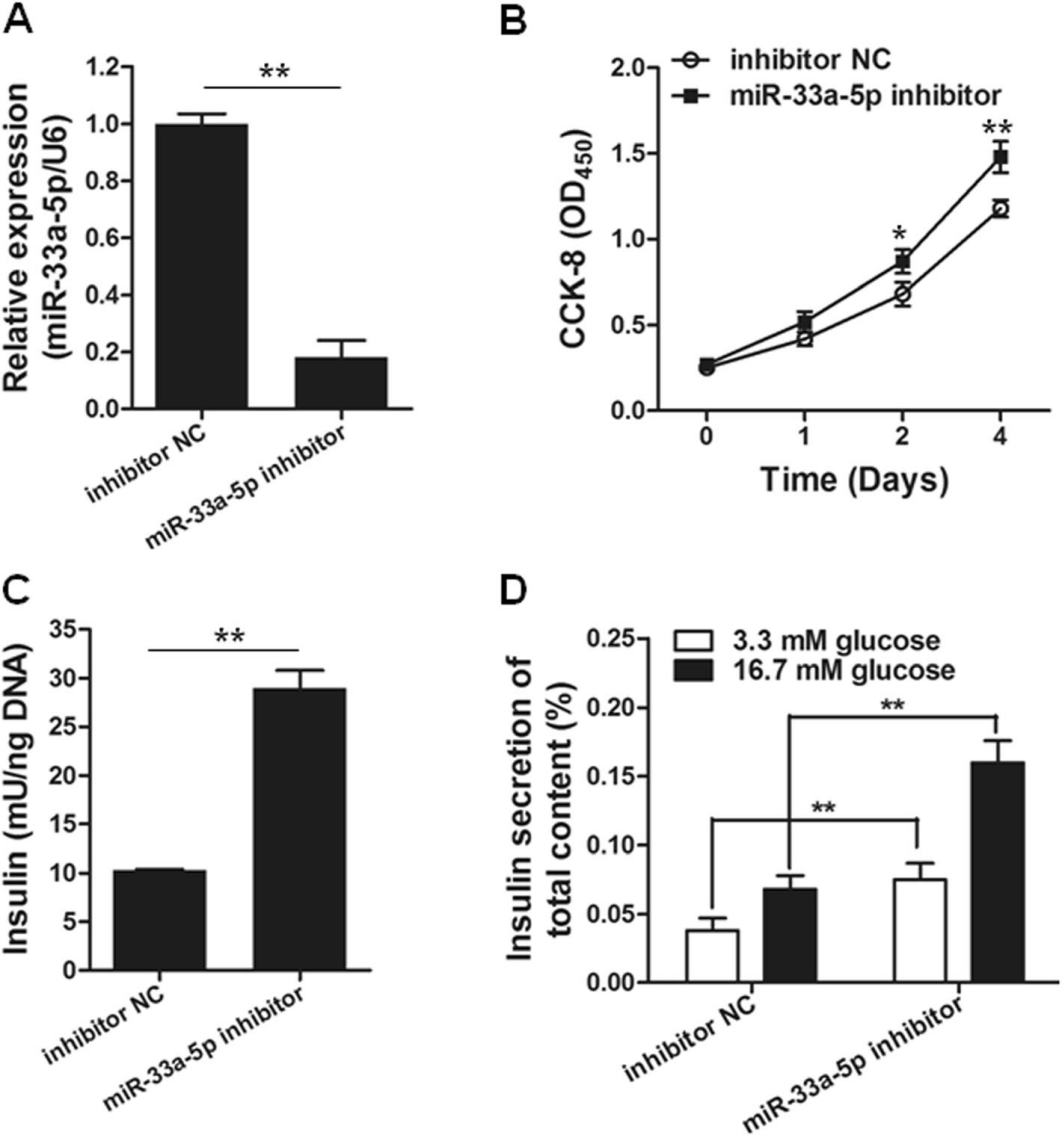
Knockdown of miR-33a-5p enhances the functions of pancreatic β cells. **a** MiR-33a-5p inhibitor and inhibitor NC were transfected into INS-1 cells for 48 h, followed by qRT-PCR. *n* = 3. **b** INS-1 cells were transfected with miR-33a-5p inhibitor or inhibitor NC for indicated time, followed by CCK-8 assay. *n* = 6 for each time point. MiR-33a-5p inhibitor and inhibitor NC were transfected into INS-1 cells for 48 h. Insulin content **(c)** and insulin secretion **(d)** were determined by ELISA assay. *n* = 6. ^*^p < 0.05, ^**^p < 0.01

To further study the function of miR-33a-5p on GDM, we knock-down of miR-33a-5p in INS-1 cells using miR-33a-5p inhibitor. The knock-down of miR-33a-5p after miR-33a-5p inhibitor transfection was confirmed by RT-qPCR assay (Fig. 3a). In contrast with the effect of miR-33a-5p overexpression on INS-1 cells, suppression of miR-33a-5p promoted the cell proliferation of INS-1 cells (Fig. 3b and c). Interestingly, inhibition of miR-33a-5p also enhanced insulin concentration and insulin secretion in both low and high glucose conditions (Fig. 3d).

### miR-33a-5p targets ABCA1

**Fig. 4:**
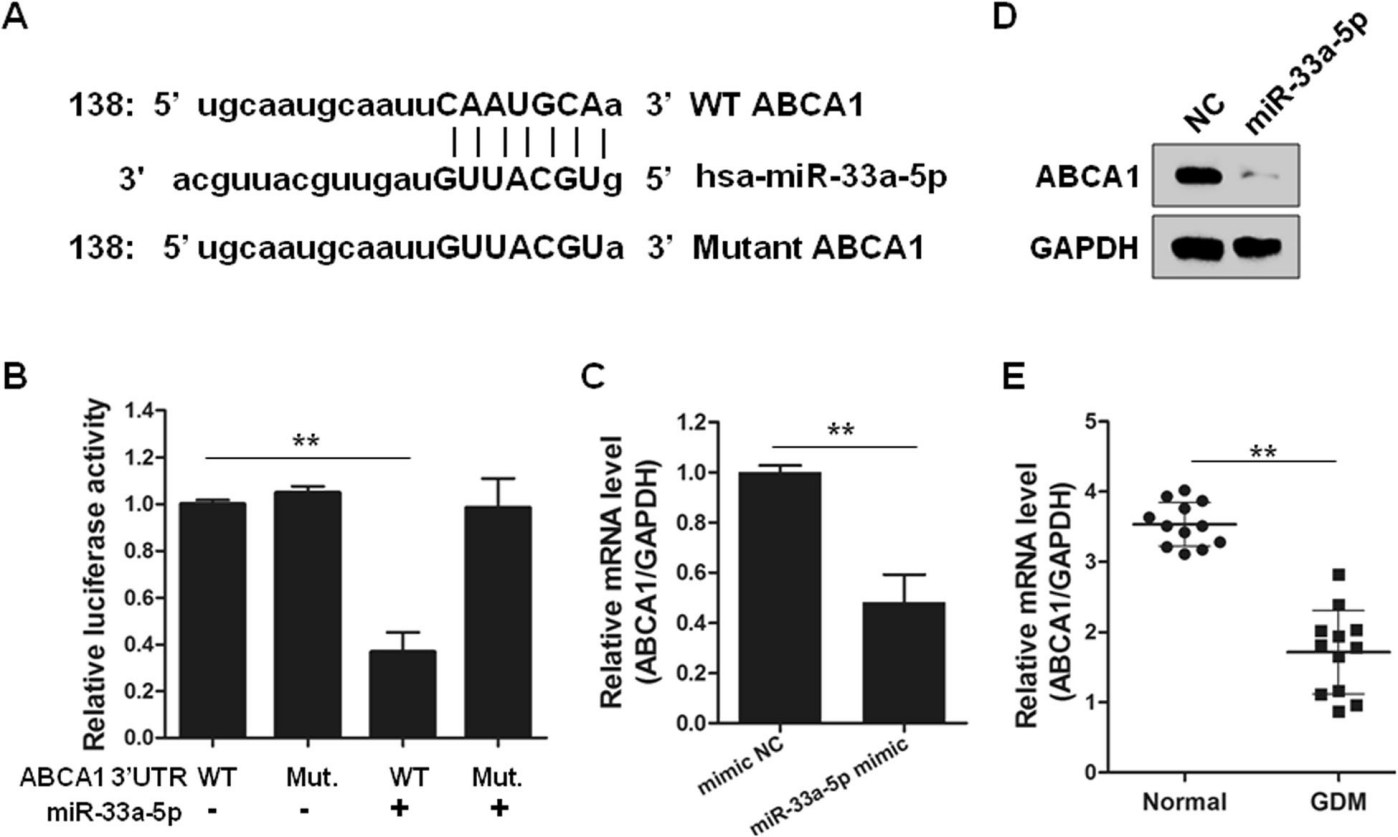
miR-33a-5p targets ABCA1 and inhibits its expression in pancreatic β cells. **a** The binding sites of miR-33a-5p in ABCA1 3’UTR was predicted online (http://www.microrna.org). **b** MiR-33a-5p mimic and mimic NC, along with wild-type (WT) or mutant (Mut.) ABCA1 3’UTR were co-transfected into HEK293T cells for 48 h, followed by luciferase assay. *n* = 3. INS-1 cells were transfected with mimic NC or miR-33a-5p mimic for 48 h, AMCA1 expressions were examined by qRT-PCR **(c)** (*n* = 3) and immunoblotting **(d)***n* = 3. **e** The expression levels of ABCA1 in peripheral blood samples from GDM pregnancies (*n* = 12) and healthy pregnancies (*n* = 12) were validated by qRT-PCR. ^**^p < 0.01

To identify a functional target of miR-33a-5p, we applied a well-established miRNA-target searching algorithm (http://www.microrna.org). We found that the 3′-UTR regions of *ABCA1* gene containing the potential binding site of miR-33a-5p (Fig. 4a). To validate ABCA1 is a target of miR-33a-5p, we applied the luciferase report, RT-qPCR, and western blot assays. We found that overexpression of miR-33a-5p specifically decreased the luciferase signal produced by the plasmid containing the wild-type, but not the mutant, 3′-UTR regions of ABCA1 in HEK293 cells (Fig. 4b). Furthermore, forced expression of miR-33a-5p reduced the expression of both mRNA and protein levels of ABCA1 in INS-1 cells (Fig. 4c and d). Finally, in contrast with miR-33a-5p, the expression levels of ABCA1 were significantly downregulated in GDM compared to normal donors (*p* < 0.01) (Fig. 4e). These results suggested that ABCA1 was a target of miR-33a-5p.

### Lnc-DANCR targets miR-33a-5p

**Fig. 5:**
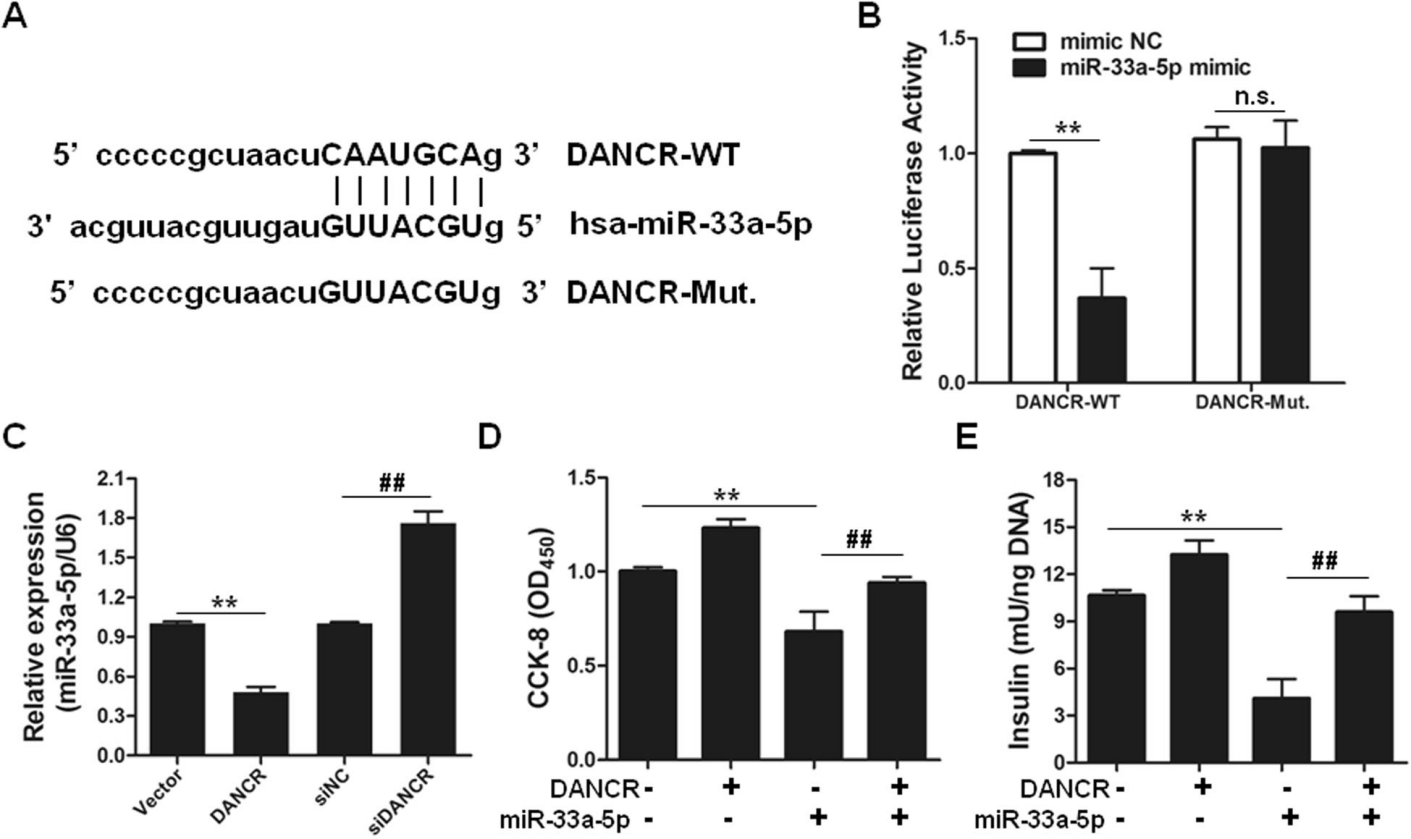
DANCR functions as a competing endogenous RNA to sponge the functions of miR-33a-5p in pancreatic β cells. **a** The predicted binding sites of miR-33a-5p and DANCR were analyzed by starBase v2.0. **b** The luciferase activity was analyzed in HEK293T cells co-transfected with wild-type DANCR (DANCR-WT) or mutated DANCR (DANCR-Mut.) and miR-33a-5p mimic or mimic NC. *n* = 3. **c** The abundance of miR-33a-5p was analyzed in INS-1 cells transfected with vector, DANCR, siNC or siDANCR. *n* = 3. **d** CCK-8 assay was measured in INS-1 cells co-transfected with DANCR, empty vector, miR-33a-5p mimic or mimic NC. *n* = 6. **e** DANCR, empty vector, miR-33a-5p mimic or mimic NC were transfected into INS-1 cells for 48 h. Insulin content was determined by ELISA assay. *n* = 6. ^**^p < 0.01, ^##^*p* < 0.01, n.s. means no significance

We have proven that miR-33a-5p targets ABCA1. However, how miR-33a-5p was regulated remains unknown. Searching the potential target lncRNA of miR-33a-5p using a well-known lncRNA-miRNA prediction tool (starBase v2.0), we identified that lnc-DANCR potentially binds with miR-33a-5p (Fig. 5a). To confirm this finding, the sequence of lnc-DANCR-WT or lnc-DANCR-Mut was inserted into the luciferase reporter plasmid. The results showed that overexpression of miR-33a-5p evidently decreased the luciferase activity of lnc-DANCR-WT, but not lnc-DANCR-Mut, suggesting that miR-33a-5p specifically binds with the sequence of lnc-DANCR-WT to reduce the luciferase signal (Fig. 5b). Indeed, lnc-DANCR overexpression reduced, whereas lnc-DANCR knock-down enhanced, the expression of miR-33a-5p in INS-1 cells (Fig. 5c).

To study the biological function of lnc-DANCR-miR-33a-5p signaling in INS-1 cells, INS-1 cells were transfected with control, lnc-DANCR, miR-33a-5p, or lnc-DANCR+miR-33a-5p combination. The results showed that lnc-DANCR upregulation promoted, whereas miR-33a-5p upregulation inhibited cell proliferation and insulin concertation of INS-1 cells. Interestingly, forced expression of lnc-DANCR can rescue miR-33a-5p-mediated inhibition effects on cell proliferation and insulin production of INS-1 cells (Fig. 5d and e).

### The correlation between lnc-DANCR, ABCA1, miR-33a-5p, and blood glucose in GDM

**Fig. 6:**
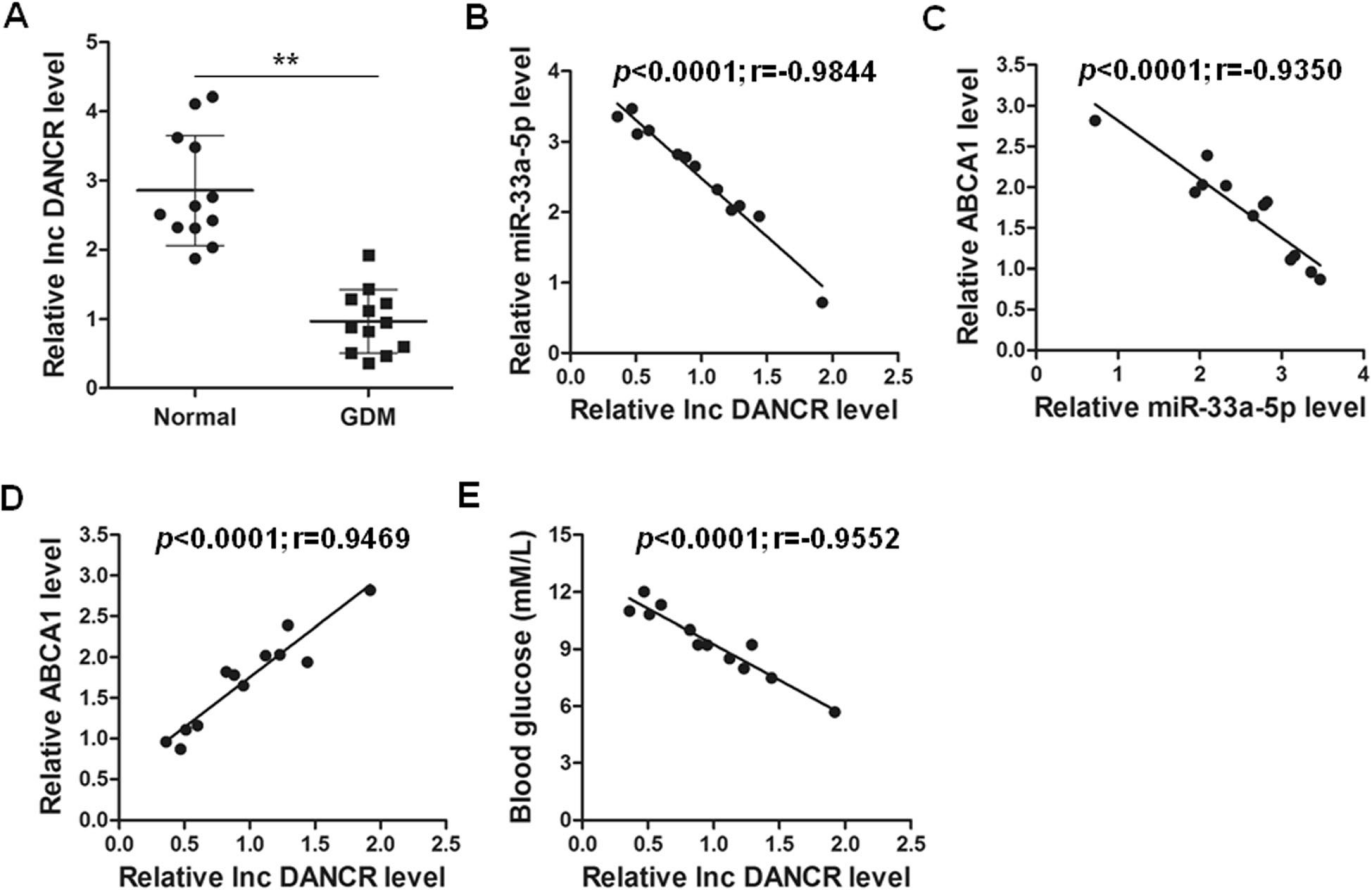
DANCR is downregulated in gestational diabetes mellitus, and negatively correlated with miR-33a-5p expression**. a** The expression levels of DANCR in peripheral blood samples from GDM pregnancies (*n* = 12) and healthy pregnancies (*n* = 12) were validated by qRT-PCR. **b-e** Correlation between miR-33a-5p level and DANCR level **(b)**, ABCA1 level and miR-33a-5p level **(c)**, ABCA1 level and DANCR level **(d)**, blood glucose level and DANCR level **(e)** were determined by Spearman correlation analysis (*n* = 15). **p < 0.01

The expression levels of lnc-DANCR in 24 blood samples from either healthy donors or GDM pregnancies were determined by RT-qPCR. The results showed that the expression levels of lnc-DANCR were significantly downregulated in GDM compared with those in healthy donors (*p* < 0.01) (Fig. 6a). We further analyzed the correlation between lnc-DANCR, ABCA1, miR-33a-5p, and blood glucose. The Spearman correlation analysis results demonstrated that a negative correlation existed between miR-33a-5p and lnc-DANCR (*p* < 0.0001; *r* = − 0.9844) (Fig. 6b), miR-33a-5p and ABCA1 (*p* < 0.0001; *r* = − 0.9350) (Fig. 6c), as well as blood glucose and lnc-DANCR (*p* < 0.0001; *r* = 0.9469) (Fig. 6d). On the contrary, a positive correlation was observed between ABCA1 and lnc-DANCR (*p* < 0.0001; *r* = − 0.9552) (Fig. 6e).

## Discussion

Dysregulation of miR-33a-5p has been identified in various human cancer, such as lung [25], prostate [26], brain [27], colon [28], and liver [29]. MiR-33a-5p regulates different targets or genes in different cancer types and exerts various effects on cancer development or progression. For example, miR-33a-5p silencing contributed to Zinc finger E-box-binding homeobox 1 (ZEB1) upregulation, and the ZEB1 promoted epithelia-mesenchymal transition (EMT) and bone metastasis of prostate cancer [26]. Downregulation of miR-33a-5p is beneficial for the chemo-resistance of lung adenocarcinoma cells via enhancing mechanistic target of rapamycin kinase (mTOR) signaling activation [30]. Moreover, overexpression of miR-33a-5p inhibited the migration and growth of colorectal cancer cells through the regulation of methylenetetrahydrofolate dehydrogenase 2 (MTHFD2), a mitochondrial enzyme involved in folic acid metabolism [28]. In addition, miR-33a-5p is also reportedly involved in the regulation of lipid metabolism [31] and inflammatory response [32]. In the present study, we, for the first time, demonstrated that miR-33a-5p was upregulated in GDM, and its level was positively correlated with blood glucose level in GDM. Of note, Li et al. reported that miR-33a-5p was downregulated in GDM compared to healthy tissues, which contradicted our findings [24]. The expression levels of miRNAs in vivo are dynamic and tissues/cells-specific. Different ways of collecting a small piece of sample from GDM or healthy placental tissues may yield different results. Thus, more work from independent labs is needed to address this question.

ABCA1 is a cholesterol efflux regulatory protein, which regulates cholesterol efflux, phospholipid homeostasis, and lipid metabolism [33, 34]. A high level of ABCA1 was observed in the endothelial cells of fetal capillaries on the maternal side of the placenta [35, 36]. ABCA1 regulates the transplacental feto-maternal lipid exchange implying its important role in normal fetal development [37]. ABCA1 was also reported to be downregulated in GDM patients [38]. Consistent with others’ publications [18–20, 38], we not only confirmed the down-regulation of ABCA1 in placental tissues of GDM patients but also identified that ABCA1 is a direct target of miR-33a-5p in GDM. We further found that the expression levels of miR-33a-5p and ABCA1 were also negatively correlated in GDM patients, which was also consistent with previous findings that the expression of miR-33a-5p and ABCA1 are inversely correlated [15–17].

The expression of miRNAs is regulated by multiple factors, such as transcription factors, epigenetic modification, miRNA biogenesis, and lncRNAs. lncRNAs often function as miRNA sponges, which is binding with miRNAs and block the ability of miRNA to bind its mRNA target [39, 40]. Lnc-DANCR is reported to be associated with the development, progression, and metastasis of various human cancers [41]. Recently studies suggested that lnc-DANCR facilitates tumor progression of glioma as well as osteosarcoma by sponging miR-33a-5p [27, 42]. Consistent with these findings, we also confirmed that lnc-DANCR bound with miR-33a-5p and reduced miR-33a-5p level in INS-1 cells. An inverse correlation was observed between lnc-DANCR and miR-33a-5p in GDM. Overexpression of lnc-DANCR facilitated cell proliferation and insulin production of INS-1 cells. More importantly, lnc-DANCR upregulation can rescue the inhibition effect of miR-33a-5p on INS-1 cells.

## Conclusion

The current study demonstrated that miR-33a-5p was upregulated in GDM. Overexpression of miR-33a-5p inhibited cell proliferation and insulin production of INS-1 cells. miR-33a-5p regulated the function of INS-1 cells may through targeting ABCA1. Lnc-DANCR downregulated miR-33a-5p via acting as a sponge of miR-33a-5p. Overexpression of Lnc-DANCR diminished the inhibition effects of miR-33a-5p on INS-1 cells. Our results provide new insight into the roles of miR-33a-5p in GDM.

## Data Availability

The datasets generated and analysed during the current study are available from the corresponding author on reasonable request.

## Abbreviations

GDM: Gestational diabetes mellitus
miRNAs: Micro RNAs
lncRNAs: Long noncoding RNAs
